# Absence of telomerase activity and telomerase catalytic subunit mRNA in melanocyte cultures

**DOI:** 10.1054/bjoc.1999.1041

**Published:** 2000-02-01

**Authors:** K Dhaene, G Vancoillie, J Lambert, J M Naeyaert, E Van Marck

**Affiliations:** Laboratory of Pathology, Department of Medicine, University of Antwerp (UIA), Universiteitsplein 1, Wilrijk–Antwerp, B-2610, Belgium; Department of Dermatology, University Hospital Gent, De Pintelaan 185, Gent, B-9000, Belgium

**Keywords:** telomerase, melanoma, hTERT, proliferation, Ki-S2, Ki-S5

## Abstract

The classic model of activation of telomerase, for which activity has been found in most cancers including cutaneous malignant melanoma (CMM), dictates that enzyme activity is generated by pathological reactivation of telomerase in telomerase-negative somatic cells. However, recent data demonstrated physiological up-regulation in some normal cell types when established as proliferating cultures, indicating that, in some cancer types, telomerase is expressed by the process of up-regulation in telomerase-competent precursor cells. In this study, cultures of epidermal melanocytes, progenitor cells of CMM, were established and harvested in the logarithmic phase of growth. Telomerase activity was looked for using a non-isotopic variant of the telomeric repeat amplification protocol, and transcript expression of the hTERT gene, the rate-limiting catalytic telomerase subunit, was investigated by the reverse transcription polymerase chain reaction. Neither telomerase activity nor hTERT mRNA could be detected in proliferating melanocyte cultures. Our in vitro data argue against the model of telomerase as a common biomarker of cell proliferation. The results further suggest that telomerase is tightly controlled in normal melanocytes, and that telomerase is reactivated rather than up-regulated in melanocytic precursors during melanoma initiation or progression. © 2000 Cancer Research Campaign


British Journal of Cancer (2000) **82**(5), 1051–1057
© 2000 Cancer Research Campaign
DOI: 10.1054/ bjoc.1999.1041, available online at http://www.idealibrary.com on IDEAL®

### Absence of telomerase activity and telomerase catalytic subunit mRNA in melanocyte cultures

**K Dhaene[1], G Vancoillie[2], J Lambert[2], JM Naeyaert[2] and E Van Marck[1]**

[1]Laboratory of Pathology, University of Antwerp (UIA), Department of Medicine, Universiteitsplein 1, B-2610 Wilrijk–Antwerp, Belgium; [2]Department of Dermatology, University Hospital Gent, De Pintelaan 185, B-9000 Gent, Belgium

**Summary** The classic model of activation of telomerase, for which activity has been found in most cancers including cutaneous malignant melanoma (CMM), dictates that enzyme activity is generated by pathological reactivation of telomerase in telomerase-negative somatic cells. However, recent data demonstrated physiological up-regulation in some normal cell types when established as proliferating cultures, indicating that, in some cancer types, telomerase is expressed by the process of up-regulation in telomerase-competent precursor cells. In this study, cultures of epidermal melanocytes, progenitor cells of CMM, were established and harvested in the logarithmic phase of growth. Telomerase activity was looked for using a non-isotopic variant of the telomeric repeat amplification protocol, and transcript expression of the hTERT gene, the rate-limiting catalytic telomerase subunit, was investigated by the reverse transcription polymerase chain reaction. Neither telomerase activity nor hTERT mRNA could be detected in proliferating melanocyte cultures. Our in vitro data argue against the model of telomerase as a common biomarker of cell proliferation. The results further suggest that telomerase is tightly controlled in normal melanocytes, and that telomerase is reactivated rather than up-regulated in melanocytic precursors during melanoma initiation or progression. © 2000 Cancer Research Campaign

**Keywords:** telomerase; melanoma; hTERT; proliferation; Ki-S2; Ki-S5

Telomeres, stretches of repetitive DNA sequences associated with specific DNA binding proteins at the ends of eukaryotic chromosomes, progressively shorten with every cell division because DNA polymerase can not replicate the end of a linear template (the ‘End-replication Problem’) (Allsopp et al, 1995; Kipling, 1995). Gradual telomere erosion has been suggested to be a ‘mitotic clock’ and tumour suppressor mechanism, triggering senescence and cell death when telomere reduction eventually destabilizes chromosomes (Harley, 1991). Acquisition of the immortal phenotype is an obligatory event for most human cancers and stabilization of telomere length is thought to be a critical molecular condition in the multistep pathway to cellular transformation and immortalization (Newbold et al, 1982; Rhyu, 1995). Telomerase is a ribonucleoprotein complex that, by the action of an internalized RNA template (hTERC), a catalytic subunit (hTERT) and a possible helper protein (hTEP1), adds telomeric DNA to the ends of chromosomes, thereby halting their erosion with each cell division (Feng et al, 1995; Harrington et al, 1997; Nakamura et al, 1997). Based on the results of the conventional telomerase polymerase assay and of the sensitive telomeric repeat amplification protocol (TRAP assay), which showed enzyme activity in over 85% of human cancer tissues and immortal cell lines but not in normal primary cell cultures or normal tissues, it was extrapolated that, putatively by a genetic event, telomerase activity is ‘turned on’ during in vivo tumorigenesis (the ‘classic’ telomerase reactivation model), thereby freeing premalignant cells from the restraint of their finite life span (Counter et al, 1992; Kim et al, 1994). However, extensive TRAP analyses and RNA expression studies of hTERT – the only rate-limiting subunit discovered today – revealed that many normal cell types too, of which the majority of cancers are derived from, are weakly telomerase-positive in vivo (Counter et al, 1995; Harle-Bachor and Boukamp, 1996; Kolquist et al, 1998; Ramakrishnan et al, 1998). Other cell types, scored telomerase-negative in vivo, appear competent to express telomerase when subjected to a sufficient proliferative stimulus in vitro (Belair et al, 1997; Greider, 1998). Therefore, it is currently only valid to apply the classic reactivation model to a particular organ system when the cell of origin is effectively telomerase-negative, not just in its in vivo state but also when subjected to ‘excessive’ growth stimulation (‘revised’ classic model or type 1 scenario) (Wynford-Thomas, 1999).

Cutaneous malignant melanoma (CMM) is a cancer originating in melanocytes, a skin-cell type derived from the neural crest (Quevedo and Fleischmann, 1980). CMM is characterized by a rapid growth rate, the invasion of local tissue and a propensity for metastasis. The incidence and mortality rates of CMM are rising dramatically throughout the world (Boyle et al, 1995). Cytogenetic and molecular studies in CMM suggest that mutations in several genes are critical in the susceptibility, development and progression of CMM, which is believed to develop in a multistep fashion (Albino, 1995). CMM evolves from melanocytic precursors via the formation of clinicopathologically defined intermediate lesions of varying stability (Briggs, 1985). Interestingly, an increase in telomerase activity has been found during progression of melanocytic lesions from melanocytic naevi to metastatic CMM, indicating that telomerase activity may play a role in tumour initiation and progression (Taylor et al, 1996; Bosserhoff et al, 1997; Glaessl et al, 1999; Parris et al, 1999) (see Table 1). However, it is

*Received 17 June 1999*
*Revised 21 October 1999*
*Accepted 25 October 1999*

*Correspondence to:* E Van Marck

**Table 1** Overview of telomerase activity (%) in melanocytic lesions, as reported in the literature

| | Taylor et al, 1996 | Bosserhoff et al, 1997 | Ueda et al, 1997 | Parris et al, 1999 | Glaessl et al, 1999 |
|---|---|---|---|---|---|
| Melanocytic naevi | – | 5/17 (29.4%) | – | – | 10/36 (27.7%) |
| Atypical naevi | – | – | – | – | 4/5 (80%) |
| Spitz's naevi | – | – | – | – | 2/3 (66%) |
| Primary CMM | 5/6 (83.3%) | 7/11 (64.0%) | 4/5 (80.0%) | 22/32 (69%) | 28/31 (90.3%) |
| CMM metastasis | 1/1 (100%) | 8/10 (80.0%) | – | – | 12/13 (92.3%) |
| CMM cell lines | – | 8/8 (100%)[a] | – | – | 8/8 (100%)[a] |

[a]Mel Im, HTZ-19, Mel Ei, Mel Wei, Mel Juso, Mel Ju, Mel Ho, SK Mel 28.

not known whether telomerase is initially de novo reactivated or merely quantitatively up-regulated during CMM carcinogenesis, because it is not known whether epidermal melanocytes are competent to express telomerase in physiological circumstances.

To investigate the competence of epidermal melanocytes to express telomerase, we determined telomerase activity and expression of hTERT, hTEP1 mRNA and hTERC RNA, using the TRAP assay and reverse transcription polymerase chain reaction (RT-PCR) respectively, in proliferating melanocyte cultures.

### MATERIALS AND METHODS

### Melanocyte cultures

Ten primary MCCs were obtained from neonatal foreskins and cultured in M199 medium supplemented with 2% fetal calf serum (FCS), $10^{-9}$ M choleratoxin, 10 ng ml$^{-1}$ basic fibroblast growth factor, 10 μg ml$^{-1}$ insulin, 1.4 μM hydrocortisone and 10 μg ml$^{-1}$ transferrin, as described previously (Naeyaert et al, 1991). Post-primary MCCs were maintained in low calcium (0.03 mM) M199 medium supplemented with the aforementioned additives and 10% FCS. Assessment of growth was performed in triplicate by cell counting using a Bürker haemocytometer and a Coulter counter, according to standard procedures. The melanocytic nature of the MCC cells was evidenced by indirect immunofluorescence using the NKIbeteb antibody (Monosan, The Netherlands) against the (pre)melanosomal silver protein, as described (Lambert et al, 1998). For analysis of telomerase activity and transcript expression, cell extracts and total RNA were collected from proliferating MCCs during logarithmic phase of growth. A precise assessment of the proliferating cell fraction in matched cytospins of these MCCs was facilitated by a streptavidin–biotin–peroxidase-based immunocytochemical approach with the monoclonal antibodies Ki-S5 and Ki-S2 (provided by Prof. Dr R Parwaresch, Institute of Haematopathology, University of Kiel, Germany). Ki-S5 binds to a formalin-resistant epitope of the Ki-67 antigen, yields identical results in fresh material and fixed tissues, and, unlike Ki-67, does not cross-react with cytoplasmic antigens of epithelial cells (Kreipe et al, 1993). Ki-S2 binds to an epitope that is present during the entire cell cycle, with exception of the rate-limiting G1 phase, thereby being a more accurate marker of the actively proliferating cell fraction than Ki-S5 (Rudolph et al, 1998). Human dermal foreskin fibroblasts, as well as G 361 melanoma cells and HL-60 cells served as telomerase-negative and -positive cells respectively.

### Non-isotopic TRAP assay

Lysate preparation and the TRAP assay were performed as described previously (Dhaene et al, 1998), with minor modifications. Briefly, $10^6$ MCC cells were lysed by retro-pipetting in 200 μl of ice-cold lysis buffer. After 30 min of incubation on ice, the lysates were centrifuged at 14 000 ***g*** for 60 min at 4°C, and the total protein concentration of the supernatant standardized according to Bradford (approximately 1 μg μl$^{-1}$). Six microlitres of supernatant were used for the TRAP assays. After an initial incubation period (30°C for 30 min), telomerase products were amplified using TS and ACX primers (35 cycles at 94°C for 30 s, 53°C for 30 s and 72°C for 30 s). Assay specificity was confirmed by inclusion of an RNAase preincubation control step, and *Taq* inhibition checked by including the 36 bp internal control. Presence of telomerase inhibitors was tested by mixing negative MCC extracts with positive HL-60 extracts in a 1:1 ratio. Every assay included a telomerase-positive sample (HL-60), a telomerase-negative sample (dermal fibroblasts) and an extract-free sample to detect PCR amplification of primer dimers. Amplicons were electrophoresed on a 12.5% non-denaturing polyacrylamide gel (19:1), stained with ethidium bromide and analysed by the CCD camera-coupled Gel Doc 1000 Molecular Analyst Software package (Bio-Rad Laboratories GmbH, Germany).

### RT-PCR analysis of telomerase transcripts

Total RNA was isolated using Tri Reagent (Sigma Chemical Co., USA). cDNAs were synthesized from 1 μg of total RNA in RT buffer containing random hexamers (Pharmacia Biotech, Sweden) and the MMLV reverse transcriptase (Promega Benelux BV, The Netherlands). For amplification of hTEP1, hTERC and hTERT transcripts, primers and cycling conditions were applied, as described previously (Dhaene et al, 1999) (see also Table 2). Briefly, primer pairs TLP1/U4792 and TLP1/L5102 (333 bp amplicon) and HTR-F and HTR-R (112 bp amplicon) were used to detect hTEP1 and hTERC cDNA. hTERT cDNA was looked for using two sets of primers. Primers LT5 and LT6 (145 bp amplicon) amplify a telomerase-specific hTERT sequence (T-motif), whereas primers TERT-2164S and TERT-2620A (457 bp amplicon) amplify two conserved reverse transcriptase motifs (A and B), thereby spanning two splice sites, which cause 36 bp and 182 bp transcript deletions (α and β splice sites respectively). During first and second hTERT cDNA amplification β-actin-specific internal control primers (95 bp) were added at 72°C of cycle 13 and 15 respectively. Amplification of genomic DNA was controlled by omitting the RT-step in appropriate control reactions.

 *© 2000 Cancer Research Campaign*

**Table 2** Oligonucleotides and cycling conditions used for detection of hTEP1, hTERC and hTERT transcripts by RT-PCR

| Oligonucleotides | C°/time (sec) | Cycles |
|---|---|---|
| hTEP1 cDNA amplification (333 bp)[a] | 94°/30 | 30 |
| TLP1/U4792: 5′-CTTGGAATTGGGTCTGGTCTCTCG-3′ | 62°/45 | |
| TLP1/L5102: 5′-CACAGCAGTAGGGGATGAGGAAAC-3′ | | |
| hTERC cDNA amplification (112 bp)[a] | 94°/30 | 25 |
| HTR-F: 5′-CCTAACTGAGAAGGGCGTAGGC-3′ | 65°/60 | |
| HTR-R: 5′-CTAGAATGAACGGTGGAAGGCG-3′ | | |
| First round hTERT cDNA amplification (145 bp)[b] | 94°/20 | |
| LT5: 5′-CGGAAGAGTGTCTGGAGCAA-3′ | 68°/40 | 33 |
| LT6: 5′-GGATGAAGCGGAGTCTGGA-3′ | 72°/30 | |
| Second round hTERT cDNA amplification (457 bp)[b] | 95°/25 | |
| TERT-2164S: 5′-GCCTGAGCTGTACTTTGTCAA-3′ | 68°/50 | 35 |
| TERT-2620A: 5′-CGCAAACAGCTTGTTCTCCATGTC-3′ | 72°/50 | |
| β-actin cDNA amplification (95 bp)[b] | (see Materials and Methods) | |
| 774: 5′-GGGAATTCAAAACTGGAACGGTGAAGG-3′ | | |
| 775: 5′-GGAAGCTTATCAAAGTCCTCGGCCACA-3′ | | |

[a]Nakayama et al (1998); [b]Ulaner et al (1998).

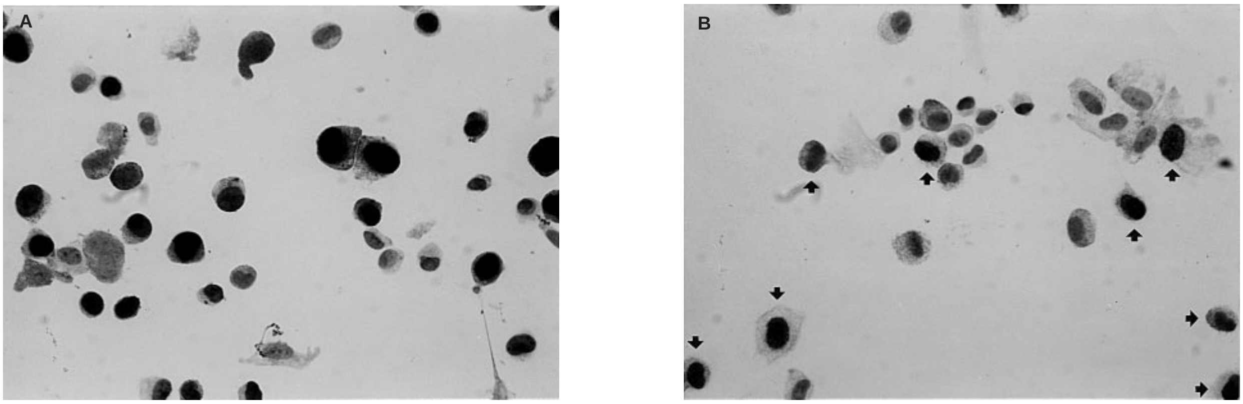

**Figure 1** Immunocytochemistry of cytospinned cultured human melanocytes (streptavidin–biotin–peroxidase technique, original magnification ×100), showing Ki-S5 staining (**A**) in 60% of nuclei, and Ki-S2 immunoreactivity (**B**) in 30% of melanocytes (arrows)

### RESULTS

Immunofluorescence with the melanosome-specific NKIbeteb antibody confirmed the melanocytic nature of cultured MCCs, with signals confined to the perinuclear area, along the dendrites and in the tips of the dendrites. The proliferative rate was found to be approximately 4.5–5 days per population doubling (data not shown). In MCCs in logarithmic phase of growth 60% and 30% of cells showed Ki-S5 and Ki-S2 nuclear staining respectively (Figure 1A,B).

A non-isotopic TRAP procedure was used to assay telomerase activity in extracts of proliferating MCCs. In a previous report, the sensitivity of our ethidium bromide-based procedure was evaluated, and 100 telomerase-positive cells were found to be sufficient for the detection of telomerase activity (Dhaene et al, 1998). In the present study, results reproducibly showed no telomerase activity in extracts of proliferating MCCs and of dermal fibroblasts (Figure 2A). False-negative results due to the presence of *Taq* DNA polymerase inhibitors or telomerase inhibitors were excluded since the internal control could be amplified and ladder signals of telomerase-positive HL-60-extracts did not disappear after mixing with telomerase-negative extracts of MCCs (Figure 2B). In extracts of G 361 melanoma cells, like with HL-60 cells, strong enzyme activity was detected.

RT-PCR, specifically amplifying transcripts of the internal RNA template hTERC, of the catalytic subunit hTERT and of an associated protein hTEP1, was used to study expression of the various components of the telomerase complex in proliferating MCCs. Under all applied cycling conditions, PCR products were not generated when omitting the RT step, making DNAase treatments unnecessary (data not shown). We detected expression of hTERC and hTEP1 in G 361 cells and in proliferating MCCs. In contrast, we observed the 145 bp hTERT RT-PCR product only in the cancer-derived cell lines but not in fibroblasts nor in any of the MCCs (Figure 3A). We further applied the primers designed by Ulaner et al (1998) that span both the β and the α splice site, causing 182 bp and 36 bp deletions respectively. PCR with the 2164/2620 primers revealed alternative splicing of the hTERT gene in all neoplastic cells (Figure 3B). Among the four amplification products, representing the full-length hTERT transcript (457 bp), the α-deleted transcript (421 bp), the β-deleted transcript (275 bp), and the α- and β-deleted transcript (239 bp), the β-deleted transcript was clearly over-represented, while the α-deleted transcript was hardly detectable. Only the full-length

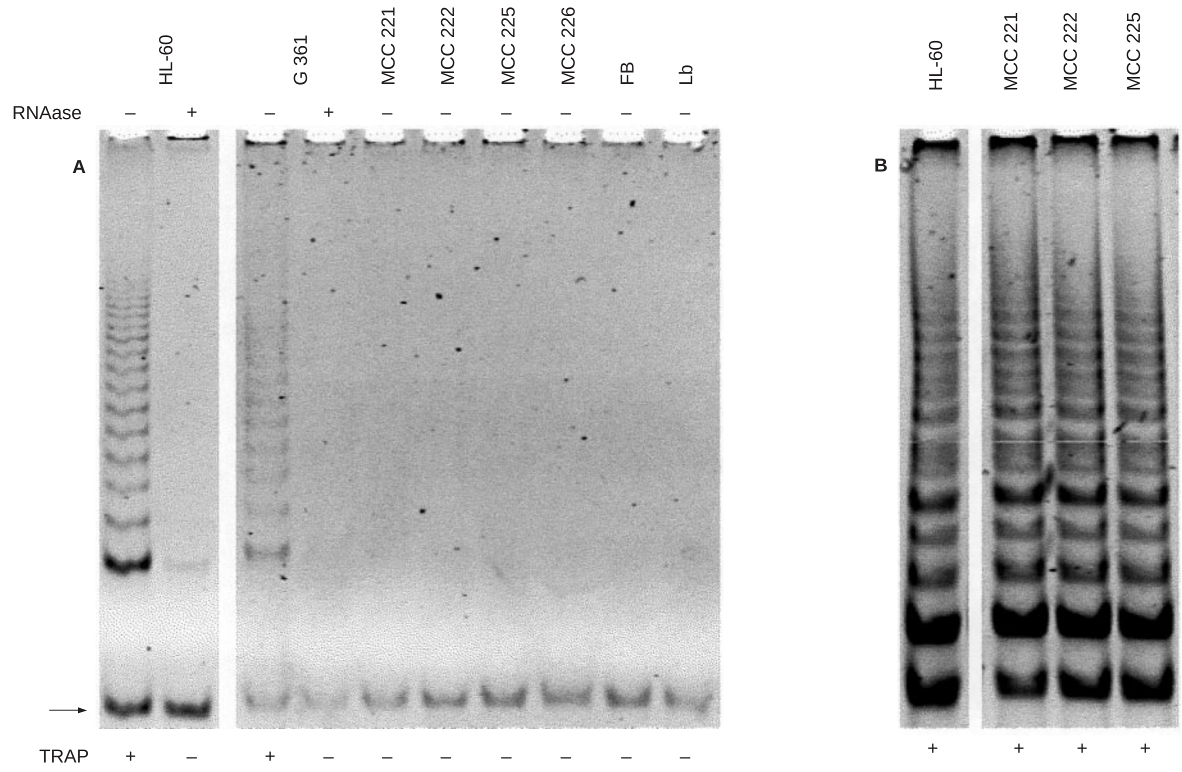

**Figure 2** Representative non-isotopic TRAP assay results of telomerase activity status in supernatant from melanocyte cultures (MCC) and G 361 melanoma cells (inverted digitized images). (**A**) Extracts of HL-60 cells and foreskin fibroblasts (FB) served as positive and negative control respectively. For every assay, a 36 bp internal control band is visible (arrow). A RNAase-treated control (+ at top), showing loss of signal, doubled each positive sample. Lysis buffer alone (Lb) was applied as another negative control. The assay result is indicated at the bottom of the figure. (**B**) Failure of the telomerase negative extracts of MCCs to inhibit the telomerase-positive HL-60 extracts in a mixing experiment. Telomerase-negative extracts of MCC 221, 222 and 225 mixed with HL-60 extract in a 1:1 ratio transcript is thought to code for active reverse transcription activity. As expected, no 457 bp amplicon but also none of the other spliced variants were detected in any of the MCCs and fibroblasts. Overall, the absence of hTERT expression in proliferating MCCs was confirmed using two different RT-PCR protocols.

### DISCUSSION

Regarding their telomerase state, normal progenitor cells of telomerase-positive tumours can be subdivided into three categories (Wynford-Thomas, 1999). The cell of origin constitutively expresses a low (Type 3), a high (Type 2) or no (Type 1) level of telomerase activity. The latter state is only acceptable when the cell type is shown to be effectively telomerase-negative when subjected to ‘excessive’ growth stimulation. De novo expression (Type 1 scenario) vs quantitative up-regulation (Type 2 and 3 scenarios) of telomerase activity, occurring during carcinogenesis, are supposed to be fundamentally distinct biological processes as to what the tightness of telomerase regulation is. This study addressed the question whether epidermal melanocytes are competent to express telomerase under proliferation-inducing conditions.

Methodologically, we had to deal with an ongoing debate. Indeed, conflicting reports have appeared concerning cell cycle-dependent regulation of telomerase activity in cancer cells and telomerase-expressing normal cells, like T lymphocytes. Most groups found that telomerase is largely absent in cells that truly exit the cell cycle ($G_o$), and that telomerase activity does not vary significantly at the other cycle stages (Mantell and Greider, 1994; Holt et al, 1996, 1997). In contrast, others concluded that $G_o$-cells have detectable telomerase, whilst maximal telomerase activity is present in S phase cells (Yamada et al, 1996; Zhu et al, 1996). To circumvent this discrepancy, and to obtain an estimation of the least possible percentage of telomerase-positive melanocytes, cytospins were stained with the monoclonal antibody Ki-S2, which excludes $G_o$-cells and cells in the rate-limiting $G_1$ phase (Rudolph et al, 1998). Thirty per cent of melanocytes showed Ki-S2 immunoreactivity, which means that, the length of the S phase equalling the lengths of both the $G_2$ and the M phase, at least $1.5 \times 10^5$ melanocytes per 200 μl CHAPS lysis buffer (4500 cells in assay) could have had detectable telomerase activity. We previously determined that telomerase activity of a minimum of $10^4$ telomerase-positive cells 200 $\mu l^{-1}$ CHAPS lysis buffer (100 cells in assay) was detected in the ethidium bromide-based TRAP assay (Dhaene et al, 1998), indicating that the observed lack of telomerase activity in proliferating MCCs reported here, was substantial, and did not result from insufficient sensitivity of the assay. Moreover, using two different RT-PCR protocols, neither full-length nor spliced hTERT transcripts were detected in any of the ten MCCs, thereby further corroborating our conclusions. Indeed, hTERT encodes the proteinaceous subunit of the telomerase complex, which, by a reverse transcriptase-like activity, catalyses the synthesis of telomeric repeats. Whereas hTERT expression can be found in the absence of telomerase activity, suggesting the occurrence of post-translational modification, telomerase activity

 *© 2000 Cancer Research Campaign*

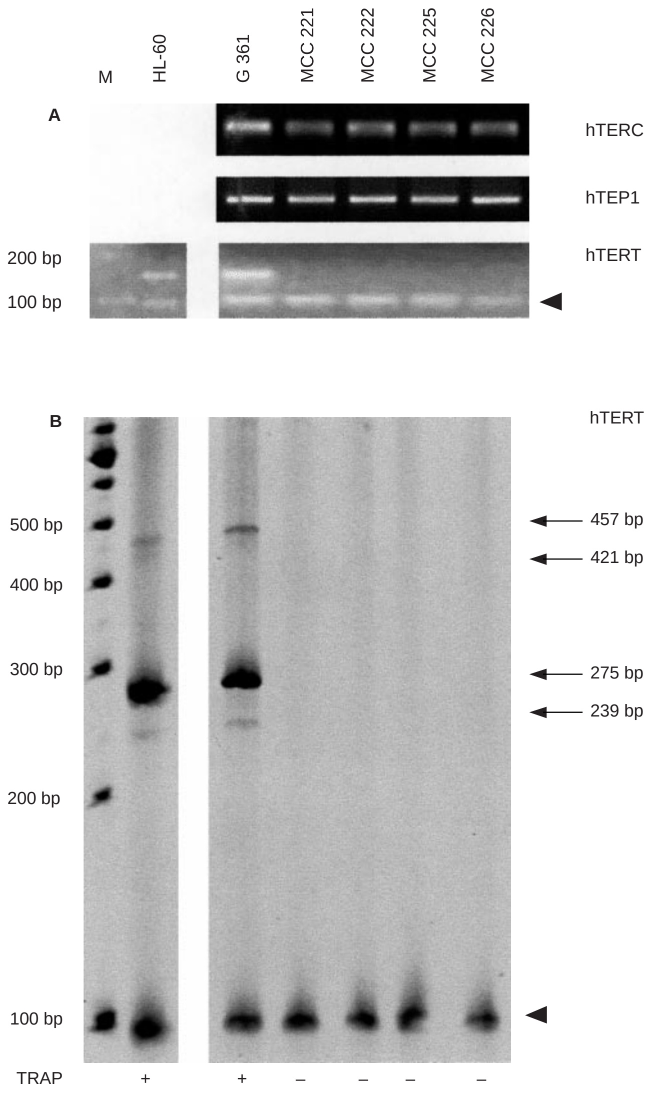

**Figure 3** (**A**) Representative results of RT-PCR analysis for the expression of hTERC, hTEP1 and hTERT in HL-60 cells (positive control), G 361 melanoma cells and cultured melanocytes (MCC). Expression of hTERT mRNA was determined using primers LT5/LT6 (145 bp amplicon). Amplicons were electrophoresed on a 2% agarose gel. (**B**) Representative results of RT-PCR analysis for the expression of hTERT using primers TERT-2164 and TERT-2620. Amplicons were electrophoresed on a 6% polyacrylamide gel (inverted digitized image). TRAP assay results for telomerase activity (bottom), the 95 bp β-actin internal control amplicon (arrowheads), and length markers (M) are indicated is always accompanied by hTERT expression (Nakamura et al, 1997). In G 361 cells, as is known for HL-60 cells, three additional transcript variants were found. However, the significance of the distribution pattern of the spliced products awaits knowledge on the role of the individual mRNA variants and elucidation whether these variants are translated into biologically functional proteins. Thus, our report adds epidermal melanocytes to the list of cell types, including fibroblasts, mammary epithelium and embryonic kidney cells, which do not express telomerase activity even when proliferating actively (Counter et al, 1992; Kim et al, 1994).

Telomerase activity has been detected in the majority of primary CMMs (range 64.0–90.3%) and CMM metastases (range 80–100%), indicating that telomerase may play a role in melanoma carcinogenesis (see Table 1). Accounting for over 50% of the cases, CMM is generally believed to derive de novo in normal skin from fully dendritic mature epidermal melanocytes (Ackerman, 1988). Thus, our data suggest that melanocytes are physiologically telomerase-incompetent, and that telomerase is pathologically reactivated during melanoma carcinogenesis. This pathogenic pathway corresponds with the aforementioned revised classic model (Type 1 scenario), in which the cell of origin is telomerase-negative, and in which telomerase reactivation is directly selected in precrisis tumour cell populations, resulting in an immortal tumour with telomeres stabilized at or above the crisis threshold (Wynford-Thomas, 1999). On the other hand, the spatial association of a subset of CMMs with benign naevi points to a possible malignant transformation of naevus cells (Hastrup et al, 1991). Whilst telomerase activity has been found to increase from benign melanocytic naevi to atypical naevi and further to CMM and metastatic CMM cells (see Table 1), both the reactivation and the up-regulation pathway are difficult to defend, since the telomerase-status of naevus cells is uncertain. It is worthwhile mentioning that it has been hypothesized that stem cells – either a pluripotential perineural cell within the neurocutaneous unit, or a committed melanoblast – are precursors of melanocytes, in both normal and abnormal differentiation, and that stem cells could be regarded as possible precursors of tumour cells (Cramer, 1991; Greaves, 1996). Stem cells are considered telomerase-competent, meaning that, if CMM would develop from it, there is no need for reactivation of telomerase. A latter scenario is currently envisaged as a Type 2 conceptual framework (Wynford-Thomas, 1999).

Mechanisms regulating telomerase reactivation are poorly understood. It is classically stated that telomerase up-regulation is forced by critical telomere erosion beyond the point where cell multiplication normally stops. An in vitro situation is seen during continuous culture of SV-40 Large T antigen or HPVE6/E7-transformed telomerase-negative human cells which eventually undergo ‘crisis’ – the condition in which cellular chromosomes are characterized by ultra-short telomeres and that coincides with telomerase activation (Wright et al, 1989; Counter et al, 1992). At present it is not known whether HPV viruses, for which DNA sequences have been found in some CMMs, can be responsible for an in vivo equivalent of crisis during CMM carcinogenesis (Scheurlen et al, 1986; Klingel et al, 1987; Takamiyagi et al, 1998).

The great majority of individual studies showed a significant positive association between incidence of CMM and high levels of intermittent solar exposure, suggesting that even a single event may suffice to stimulate tumour growth (Elwood and Jopson, 1997). All the evidence suggests that it is the UV portion of the solar spectrum which is relevant. However, the contribution of specific wavelength bands (290–320 nm for UVB and 320–400 nm for UVA radiation) and the action spectrum for melanoma induction in humans remains unknown (International Agency for Research on Cancer, 1992). It has been reported that telomerase is activated during radiation-induced malignant transformation of human cells and in mouse skin (Pandita et al, 1996; Balasubramanian et al, 1999), and in the sun-exposed skin in humans, indicating a possible modulation of telomerase activity by UV exposure (Taylor et al, 1996; Ueda et al, 1997). The prediction

© 2000 Cancer Research Campaign is that one or more tumour suppressor genes prevent activation of telomerase in normal human cells (deLange, 1998). Therefore, it needs further study to find out if UV can directly eliminate such genes, for which candidates are thought to locate on the short arm of chromosome 3 (Cuthbert et al, 1999). Alternatively, telomerase reactivation might be an epiphenomenon of UV light-induced genotoxic stress, as proposed by adherents of the 'co-selection hypothesis' (Kipling, 1997). In this report, we excluded that proliferative behaviour, which both in vitro and in vivo is another effect of UV light (Libow et al, 1988; Gilchrest et al, 1998), can co-select telomerase activity. Finally, recent cloning and sequence analysis of the hTERT gene promoter revealed the presence of binding sites for transcription factors including the c-*Myc* proto-oncoprotein (Cong et al, 1999). The latter activates telomerase by inducing expression of its catalytic subunit, indicating that hTERT is a target of c-*Myc* activity (Wu et al, 1999). Interestingly, altered expression of c-*myc* has been reported in both cultured melanoma cells and in tumour samples (Weterman et al, 1994).

### ACKNOWLEDGEMENTS

Foreskin samples were provided by the Departments of Urology and Plastic Surgery of the 'Saint-Lucas' Hospital Gent and the University Hospital Gent, respectively. The authors are grateful to Martine De Mil for establishing and maintaining the melanocyte cultures. Ki-S2 and Ki-S5 antibodies were a generous gift from Prof. Dr R Parwaresch from the Institute of Haematopathology, Kiel, Germany.

### REFERENCES

Ackerman AB (1988) What naevus is dysplastic, a syndrome and the commonest precursor of malignant melanoma? A riddle and an answer. *Histopathology* **13**: 241–256

Albino AP (1995) Genes involved in melanoma susceptibility and progression. *Curr Opin Oncol* **7**: 162–169

Allsopp RC, Chang E, Kashefi AM, Rogaev EI, Piatyszek MA, Shay JW and Harley CB (1995) Telomere shortening is associated with cell division in vitro and in vivo. *Exp Cell Res* **220**: 194–200

Balasubramanian S, Kim KH, Ahmad N and Mukhtar H (1999) Activation of telomerase and its association with G1-phase of the cell cycle during UVB-induced skin tumorigenesis in SKH-1 hairless mouse. *Oncogene* **18**: 1297–1302

Belair CD, Yeager TR, Lopez PM and Reznikoff CA (1997) Telomerase activity: a biomarker of cell proliferation, not malignant transformation. *Proc Natl Acad Sci USA* **94**: 13677–13682

Bosserhoff AK, Glaessl A, Stolz W and Buettner R (1997) Detection of telomerase activity in skin, melanocytic naevi, and melanoma by telomerase PCR ELISA. *Biochemica* **3**: 16–18

Boyle P, Maisonneuve P and Doré J (1995) Epidemiology of malignant melanoma. *Br Med Bull* **51**: 523–547

Briggs JC (1985) Melanoma precursor lesions and borderline melanomas. *Histopathology* **9**: 1251–1262

Cong YS, Wen JP and Bacchetti S (1999) The human telomerase catalytic subunit hTERT: organization of the gene and characterization of the promoter. *Hum Mol Genet* **8**: 137–142

Counter CM, Avilion AA, LeFeuvre CE, Stewart NG, Greider CW, Harley CB and Bacchetti S (1992) Telomere shortening associated with chromosome instability is arrested in immortal cells which express telomerase activity. *EMBO J* **11**: 1921–1929

Counter CM, Gupta J, Harley CB, Leber B and Bacchetti S (1995) Telomerase activity in normal leucocytes and in hematologic malignancies. *Blood* **85**: 2315–2320

Cramer SF (1991) The origin of epidermal melanocytes. *Arch Pathol Lab Med* **115**: 115–119

Cuthbert AP, Bond J, Trott DA, Gill S, Broni J, Marriott A, Khoudoli G, Parkinson EK, Cooper CS and Newbold RF (1999) Telomerase repressor sequences on chromosome 3 and induction of permanent growth arrest in human breast cancer cells. *J Natl Cancer Inst* **91**: 37–45

deLange T (1998) Telomeres and senescence: ending the debate. *Science* **279**: 334–335

Dhaene K, Hübner R, Kumar-Singh S, Weyn B and Van Marck E (1998) Telomerase activity in human pleural mesothelioma. *Thorax* **53**: 915–918

Dhaene K, Wauters J, Weyn B, Timmermans J-P and Van Marck E (1999) Expression profile of telomerase subunits in human pleural mesothelioma. *J Pathol* (in press)

Elwood JM and Jopson J (1997) Melanoma and sun exposure: an overview of published studies. *Int J Cancer* **73**: 198–203

Feng J, Funk WD, Wang SS, Weinrich SL, Avilion AA, Chiu CP, Adams RR, Chang E, Allsopp RC, Yu J, Le S, West MD, Harley CB, Andrews WH, Greider CW and Villeponteau B (1995) The RNA component of human telomerase. *Science* **269**: 1236–1241

Gilchrest BA, Park H-Y, Eller M and Yaar M (1998) The photobiology of the tanning response. In: *The Pigmentary System: Physiology and Pathophysiology*, Nordlund JJ, Boissy RE, Hearing VJ, King RA and Ortonne J-P (eds), pp. 359–372. Oxford University Press: New York

Glaessl A, Bosserhoff AK, Buettner R, Hohenleutner U, Landthaler M and Stolz W (1999) Increase in telomerase activity during progression of melanocytic cells from melanocytic naevi to malignant melanomas. *Arch Dermatol Res* **291**: 81–87

Greaves M (1996) Is telomerase activity in cancer due to selection of stem cells and differentiation arrest. *Trends Genet* **12**: 127–128

Greider CW (1998) Telomerase activity, cell proliferation, and cancer. *Proc Natl Acad Sci USA* **95**: 90–92

Harle-Bachor C and Boukamp P (1996) Telomerase activity in the regenerative basal layer of the epidermis inhuman skin and in immortal and carcinoma-derived skin keratinocytes. *Proc Natl Acad Sci USA* **93**: 6476–6481

Harley CB (1991) Telomere loss: mitotic clock or genetic time bomb. *Mutat Res* **256**: 271–282

Harrington L, McPhail T, Mar V, Zhou W, Oulton R, Bass MB, Arruda I and Robinson MO (1997) A mammalian telomerase-associated protein. *Science* **275**: 973–977

Hastrup N, Osterlind A, Drzewiecki KT and Hou-Jensen K (1991) The presence of dysplastic nevus remnants in malignant melanomas. A population-based study of 551 malignant melanomas. *Am J Dermatopathol* **13**: 378–385

Holt SE, Wright WE and Shay JW (1996) Regulation of telomerase activity in immortal cell lines. *Mol Cell Biol* **16**: 2932–2939

Holt SE, Aisner DL, Shay JW and Wright WE (1997) Lack of cell cycle regulation of telomerase activity in human cells. *Proc Natl Acad Sci USA* **94**: 10687–10692

International Agency for Research on Cancer (1992) *IARC monographs on the Evaluation of Carcinogenic Risks to Humans: Ultraviolet Radiation*, Vol. 55 IARC: Lyon

Kim NW, Piatyszek MA, Prowse KR, Harley CB, West MD, Ho PL, Coviello GM, Wright WE, Weinrich SL and Shay JW (1994) Specific association of human telomerase activity with immortal cells and cancer. *Science* **266**: 2011–2015

Kipling D (1995) *The Telomere*. Oxford University Press: Oxford

Kipling D (1997) Telomere structure and telomerase expression during mouse development and tumorigenesis. *Eur J Cancer* **33**: 792–800

Klingel R, Mincheva A, Kahn T, Gissmann L, Dippold W, Meyer zum Buschenfelde KH and zur Hausen H (1987) An amplification unit in human melanoma cells showing partial homology with sequences of human papillomavirus type 9 and with nuclear antigen 1 of the Epstein–Barr virus. *Cancer Res* **47**: 4485–4492

Kolquist KA, Ellisen LW, Counter CM, Meyerson M, Tan LK, Weinberg RA, Haber DA and Gerald WL (1998) Expression of TERT in early premalignant lesions and a subset of cells in normal tissues. *Nat Genet* **19**: 182–186

Kreipe H, Wacker HH, Heidebrecht H-J, Haas K, Hauberg M, Tiemann M and Parwaresch R (1993) Determination of the growth fraction in non-Hodgkin's lymphomas by monoclonal antibody Ki-S5 directed against a formalin-resistant epitope of the Ki-67 antigen. *Am J Pathol* **142**: 1689–1694

Lambert J, Onderwater J, Vander Haeghen Y, Vancoillie G, Koerten HK, Mommaas AM and Naeyaert JM (1998) Myosin V colocalises with melanosomes and subcortical actin bundles not associated with stress fibers in human epidermal melanocytes. *J Invest Dermatol* **111**: 835–840

Libow LF, Scheide S and DeLeo VA (1988) Ultraviolet radiation acts as an independent mitogen for normal human melanocytes in culture. *Pigment Cell Res* **1**: 397–401

Mantell LL and Greider CW (1994) Telomerase activity in germline and embryonic cells of Xenopus. *EMBO J* **13**: 3211–3217

 *© 2000 Cancer Research Campaign*

Naeyaert JM, Eller M, Gordon PR, Park H-Y and Gilchrest BA (1991) Pigment content of cultured human melanocytes does not correlate with tyrosinase message level. *Br J Dermatol* **125**: 297–303

Nakamura TM, Morin GB, Chapman KB, Weinrich SL, Andrews WH, Lingner J, Harley CB and Cech TR (1997) Telomerase catalytic subunit homologs from fission yeast and human. *Science* **277**: 955–959

Nakayama J, Tahara H, Tahara E, Saito M, Ito K, Nakamura H, Nakanishi T, Ide T and Ishikawa F (1998) Telomerase activation by hTRT in human normal fibroblasts and hepatocellular carcinomas. *Nat Genet* **18**: 65–68

Newbold RF, Overell RW and Connell JR (1982) Induction of immortality is an early event in malignant transformation of mammalian cells by carcinogens. *Nature* **299**: 633–635

Pandita TK, Hall EJ, Hei TK, Piatyszek MA, Wright WE, Piao CQ, Pandita RK, Willey JC, Geard CR, Kastan MB and Shay JW (1996) Chromosome end-to-end associations and telomerase activity during cancer progression in human cells after treatment with alpha-particles simulating radon progeny. *Oncogene* **13**: 1423–1430

Parris CN, Jezzard S, Silver A, MacKie R, McGregor JM and Newbold RF (1999) Telomerase activity in melanoma and non-melanoma skin cancer. *Br J Cancer* **79**: 47–53

Quevedo WC and Fleischmann RD (1980) Developmental biology of mammalian melanocytes. *J Invest Dermatol* **75**: 116–120

Ramakrishnan S, Eppenberger U, Mueller H, Shinkai Y and Narayanan R (1998) Expression profile of the putative catalytic subunit of the telomerase gene. *Cancer Res* **58**: 622–625

Rhyu MS (1995) Telomeres, telomerase, and immortality. *J Natl Cancer Inst* **87**: 884–894

Rudolph P, Knüchel R, Endl E, Heidebrecht H-J, Hofstädter F and Parwaresch R (1998) The immunohistochemical marker Ki-S2: cell cycle kinetics and tissue distribution of a novel proliferation-specific antigen. *Mod Pathol* **11**: 450–456

Scheurlen W, Gissmann L, Gross G and zur Hausen H (1986) Molecular cloning of two new HPV types (HPV 37 and HPV 38) from keratoacanthoma and a malignant melanoma. *Int J Cancer* **37**: 505–510

Takamiyagi A, Asato T, Nakashima Y and Nonaka S (1998) Association of human papillomavirus type 16 with malignant melanoma. *Am J Dermatopathol* **20**: 69–73

Taylor RS, Ramirez RD, Ogoshi M, Chaffins M, Piatyszek MA and Shay JW (1996) Detection of telomerase activity in malignant and nonmalignant skin conditions. *J Invest Dermatol* **106**: 759–765

Ueda M, Ouhtit A, Bito T, Nakazawa K, Lübbe J, Ichihashi M, Yamasaki H, and Nakazawa H (1997) Evidence for UV-associated activation of telomerase in human skin. *Cancer Res* **57**: 370–374

Ulaner GA, Hu JF, Vu TH, Giudice LC and Hoffman AR (1998) Telomerase activity in human development is regulated by human telomerase reverse transcriptase (hTERT) transcription and by alternate splicing of hTERT transcripts. *Cancer Res* **58**: 4168–4172

Weterman MA, Van Muijen GN, Bloemers HP and Ruiter DJ (1994) Molecular markers of melanocytic tumor progression. *Lab Invest* **70**: 593–608

Wright WE, Pereira SO and Shay JW (1989) Reversible cellular senescence: implications for immortalization of normal human diploid fibroblasts. *Mol Cell Biol* **9**: 3088–3092

Wu K, Grandori C, Amacker M, Simon-Vermot N, Polack A, Lingner J and Dallafavera R (1999) Direct activation of TERT transcription by c-Myc. *Nat Genet* **21**: 220–224

Wynford-Thomas D (1999) Cellular senescence and cancer. *J Pathol* **187**: 100–111

Yamada O, Motoji T and Mizoguchi H (1996) Up-regulation of telomerase activity in human lymphocytes. *Biochim Biophys Acta* **1314**: 260–266

Zhu X, Kumar R, Mandal M, Sharma HW, Dhingra U, Sokoloski JA, Hsiao R and Narayanan R (1996) Cell cycle-dependent modulation of telomerase activity in tumor cells. *Proc Natl Acad Sci USA* **93**: 6091–6095

*© 2000 Cancer Research Campaign*
